# Weighted Gene Co-expression Network Analysis (WGCNA) of Wnt Signaling Related to Periodontal Ligament Formation: A Bioinformatics-Based Analysis

**DOI:** 10.7759/cureus.63639

**Published:** 2024-07-02

**Authors:** Pradeep Kumar Yadalam, Ramya Ramadoss, Deepavalli Arumuganainar

**Affiliations:** 1 Department of Periodontics, Saveetha Dental College and Hospitals, Saveetha Institute of Medical and Technical Sciences, Saveetha University, Chennai, IND; 2 Department of Oral Pathology and Oral Biology, Saveetha Dental College and Hospitals, Saveetha Institute of Medical and Technical Sciences, Saveetha University, Chennai, IND

**Keywords:** regeneration, hub gene, wnt signalling, wgcna, periodontal ligament

## Abstract

Introduction

The Wnt signaling pathway is crucial for tooth development, odontoblast differentiation, and dentin formation. It interacts with epithelial cadherin (E-cadherin) and beta-catenin in tooth development and periodontal ligament (PDL) formation. Dysregulation of Wnt signaling is linked to periodontal diseases, requiring an understanding of therapeutic interventions. Weighted gene co-expression network analysis (WGCNA) can identify co-expressed gene modules. Our study aims to identify hub genes in WGCNA analysis of Wnt signaling-based PDL formation.

Methods

The study used a microarray dataset GSE201313 from the National Center for Biotechnology Information (NCBI) Gene Expression Omnibus to analyze the impact of DMP1 expression on XLH dental pulp cell differentiation and PDL formation. The standardized dataset was used for WGCNA analysis, which generated a co-expression network by calculating pairwise correlations between genes and constructing an adjacency matrix. The topological overlap matrix (TOM) was transformed into a hierarchical clustering tree and then cut into modules or clusters of highly interconnected genes. The module eigengene (ME) was calculated for each module, and the genes within this module were identified as hub genes. Gene ontology (GO) and KEGG pathway enrichment analysis were performed to gain insights into the biological functions of the hub genes. The integrated Differential Expression and Pathway analysis (iDEP) tool (http://bioinformatics.sdstate.edu/idep/; South Dakota State University, Brookings, USA) was used for WGCNA analysis.

Results

The study used the WGCNA package to analyze 1,000 differentially expressed genes, constructing a gene co-expression network and generating a hierarchical clustering tree and TOM. The analysis reveals a scale-free topology fitting index R2 and mean connectivity for various soft threshold powers, with an R2 value of 5. COL6A1, MMP3, BGN, COL1A2, and FBN2 are hub genes implicated in PDL development.

Conclusion

The study identified key hub genes, including COL6A1, MMP3, BGN, and FBN2, crucial for PDL formation, tissue remodeling, and cell-matrix interactions, guiding future therapeutic strategies.

## Introduction

Odontoblast differentiation and tooth development are intricately linked with activating the Wnt signaling pathway, which plays a crucial role in tooth morphogenesis and dentin formation [1]. Despite decreased Wnt signaling with age, stabilizing beta-catenin (β-catenin) in the adult pulp can lead to dentin formation [2,3]. The interaction between structural changes during tooth development and cell signaling is crucial, with epithelial cadherin (E-cadherin) and β-catenin playing key roles in odontoblast differentiation. Previous research explores the role of Wnt signaling in dental pulp cells, its effects on tooth development, and the role of inhibitors. It also discusses the temporal regulation of Wnt pathways and their implications for understanding diseases like XLH. Previous study discusses the role of E-cadherin and β-catenin in adherens junctions and their regulation by the Wnt pathway during palisade formation. It highlights how β-catenin and E-cadherin protein levels change with differentiation induction in different cell types, particularly DMP1 status [4].

The Wnt signaling pathway is crucial in periodontal ligament (PDL) formation, regulating cell processes during development and tissue homeostasis. It plays a role in the differentiation, maturation, and recruitment of stem cells and in establishing the PDL's unique architecture [1,5,6]. The pathway stabilizes and nuclear translocates β-catenin, a transcriptional co-activator, and inhibits the destruction complex. It also promotes the differentiation of dental follicle cells into PDL fibroblasts, promoting their migration and proliferation towards the PDL. The Wnt pathway also establishes the PDL's unique architecture and organization, regulating the attachment of PDL fibroblasts to the tooth root surface and the formation of Sharpey's fibers. Dysregulation of Wnt signaling has been linked to periodontal diseases, emphasizing the need to understand this pathway's role in PDL biology for potential therapeutic interventions [7].

Weighted gene co-expression network analysis (WGCNA) is a bioinformatics tool that identifies gene modules or networks where genes share similar expression patterns. It differs from traditional methods like differential gene expression analysis, focusing on individual genes [8]. WGCNA constructs a gene coexpression network, allowing for the identification of modules with similar expression patterns. It assigns a module, eigengene, representing the overall expression profile of each module, which can be correlated with phenotypic data or clinical parameters. WGCNA also identifies hub genes, which are key regulators or drivers of the module, providing insights into molecular mechanisms and potential therapeutic targets [9].

The Wnt signaling pathway regulates cell proliferation, differentiation, and tissue patterning for tooth development [10-12]. It plays a role in dental epithelial cell fate determination, tooth germ formation, tooth morphogenesis, and dental stem cell maintenance [13]. A comprehensive analysis method like WGCNA can be employed to understand the molecular mechanisms underlying Wnt signaling in odontogenic tissue formation. This method can identify co-expressed gene modules and potential hub genes, which may be critical players in odontogenic tissue formation [8,9,14]. Functional enrichment analysis can provide insights into the biological processes and pathways associated with Wnt signaling in odontogenesis. Our study aims to identify hub genes in WGCNA analysis of Wnt signaling-based PDL formation.

## Materials and methods

Data retrieval

The microarray dataset GSE201313 from the National Center for Biotechnology Information (NCBI) Gene Expression Omnibus was selected for analysis. The dataset investigates the impact of DMP1 expression on XLH dental pulp cell differentiation using RNA sequencing after inducing odontogenic differentiation with different phosphate sources. We retrieved it for WGCNA analysis for PDL formation.

iDEP tool

The standardized dataset was used as input for further analysis using the integrated Differential Expression and Pathway analysis (iDEP) tool (http://bioinformatics.sdstate.edu/idep/; South Dakota State University, Brookings, USA). The iDEP tool [15] is a bioinformatics platform that provides various analyses for gene expression data.

Data retrieval and preprocessing

The microarray dataset GSE228306 from the NCBI Gene Expression Omnibus was selected for analysis. The expression data was downloaded and imported into the iDEP tool.

WGCNA module detection

The standardized gene expression dataset was used as input for WGCNA analysis [16] in iDEP. WGCNA generates a co-expression network by calculating pairwise correlations between genes and constructing an adjacency matrix. The adjacency matrix was transformed into a topological overlap matrix (TOM), measuring genes' interconnectedness. Using the average linkage method, the TOM was created to create a hierarchical clustering tree (dendrogram). The tree was cut into modules or clusters of highly interconnected genes using the dynamic tree-cutting algorithm with a minimum module size of 30.

Identification of hub genes

The module eigengene (ME) was calculated for each module, which represents the first principal component of gene expression within a module. The MEs were correlated with external traits or conditions to identify modules associated with the traits of interest. The module with the highest correlation to the trait of interest was considered the hub module, and the genes within this module were considered hub genes.

Functional enrichment analysis

Gene ontology (GO) analysis and pathway enrichment analysis were performed in iDEP to gain insights into the biological functions of the hub genes. GO analysis identifies the functional categories (biological processes, molecular functions, and cellular components) significantly enriched among the hub genes. KEGG (Kyoto Encyclopedia of Genes and Genomes) pathway enrichment analysis identifies the pathways significantly enriched among the hub genes. The significance of enrichment was calculated using hypergeometric tests, and a p-value threshold (<0.05) was applied to determine the significantly enriched GO terms and KEGG pathways.

The iDEP tool offers visualization tools for WGCNA analysis, including heatmaps, clustering dendrograms, and network graphs. These tools highlight hub genes and their connections in the co-expression network, allowing for a visual representation of interconnectivity and functional relationships. The methods are specific to WGCNA analysis, and more information is recommended.

## Results

This analysis showed closely related genes are grouped into modules, and colored lines designate which modules each gene belongs to. The study created a gene co-expression network using the WGCNA program to analyze 1,000 genes that showed differential expression. A soft thresholding power of 5 was selected to guarantee a scale-free topology, resulting in the formation of a hierarchical clustering tree. A TOM was created to allow for a more thorough examination of the network architecture.

COL6A1, MMP3, BGN, COL1A2, and FBN2 genes are implicated in PDL development. The genes COL6A1, MMP3, BGN, COL1A2, and FBN2 are essential for maintaining structural integrity and remodeling. COL6A1 encodes collagen type VI, while MMP3 is involved in matrix metalloproteinase 3 (MMP-3). BGN regulates cell adhesion, migration, and proliferation. COL1A2 is a collagen type I protein crucial for PDL development. FBN2 is a glycoprotein that forms and maintains elastic fibers in connective tissues.

The WGCNA uses a dendrogram to visually represent the hierarchical clustering of genes based on their expression patterns. This process helps identify distinct modules or groups of genes with similar expression profiles. The dendrogram is represented in diverse hues or colors, with genes within the same module having similar expression patterns. This intuitive method helps researchers analyze complex gene expression data and identify gene networks involved in specific biological processes or pathways.

**Figure 1:**
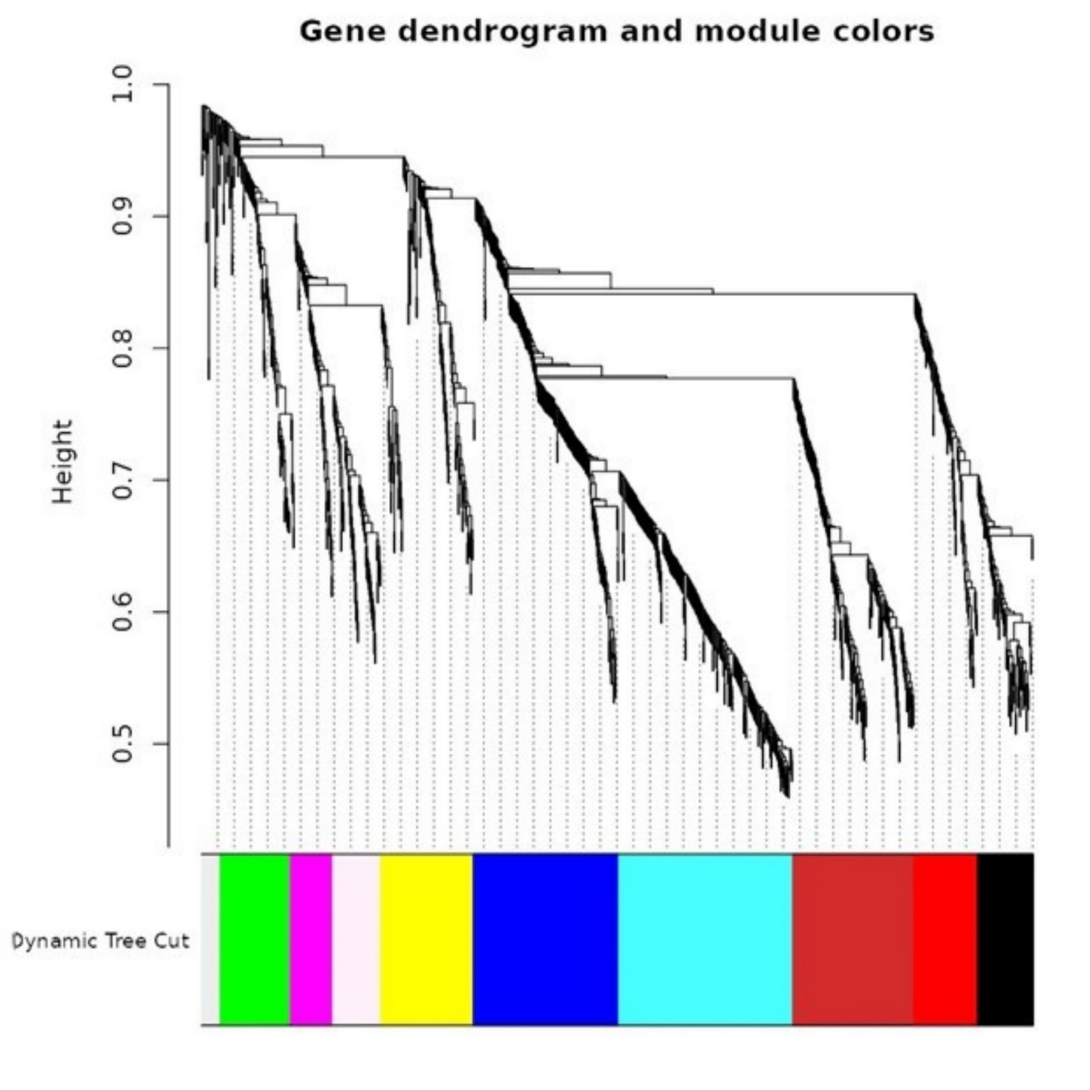
The image shows a dendrogram formed by applying dissimilarity clustering techniques visually representing distinct modules in diverse hues.

**Figure 2:**
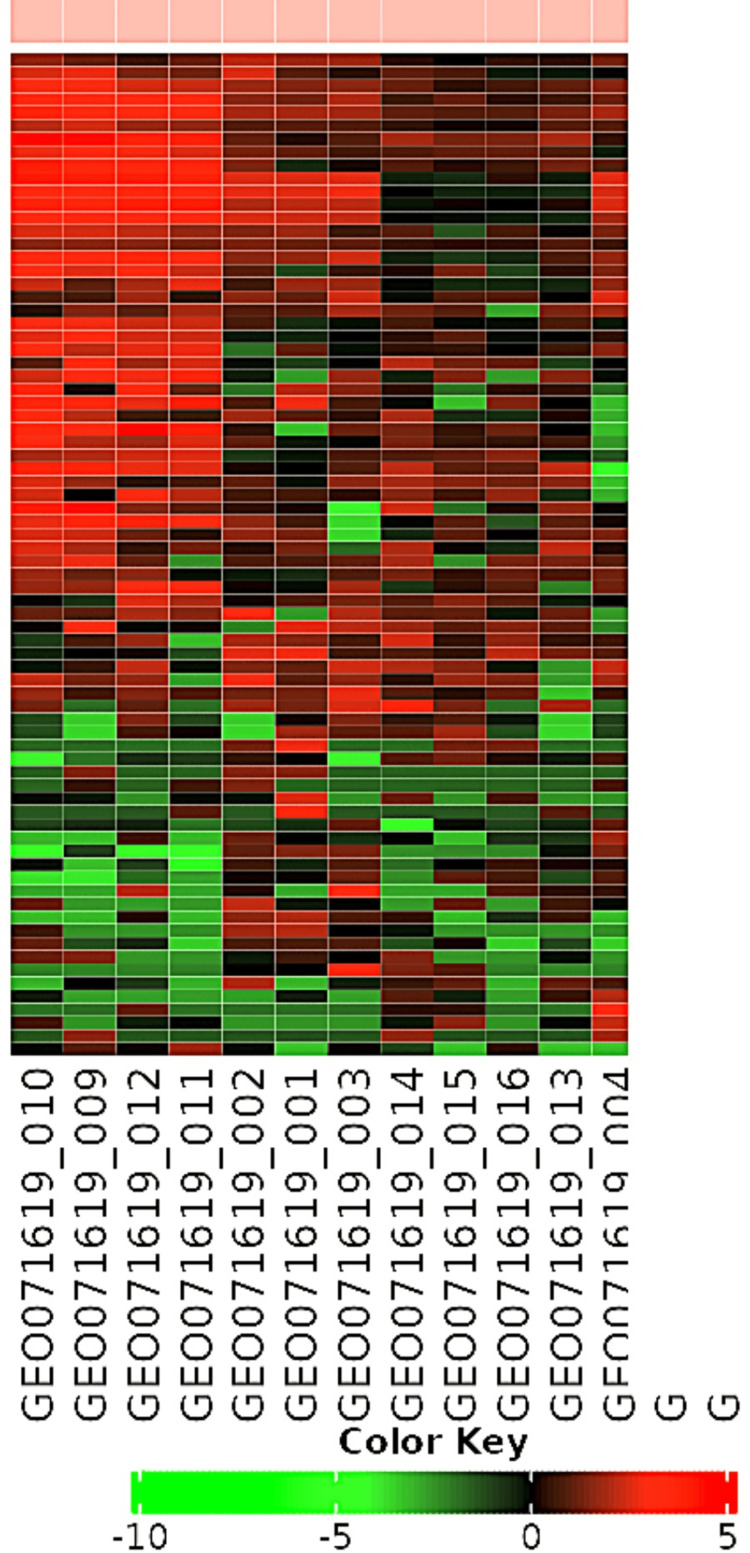
The image shows the heat map graphic and the topological overlap matrix of all the genes analyzed.

WGCNA analysis uses a heat map graphic to visualize gene expression patterns across different samples or conditions. This color-coded matrix helps identify genes with similar or distinct profiles, allowing for coexpression or differential expression identification. The TOM quantifies the relationship between genes based on their co-expression patterns, forming a dendrogram or hierarchical clustering tree. This combination of the heat map and dendrogram helps characterize and interpret gene co-expression networks in the context of WGCNA analysis as explained in Figures 1-2.

**Figure 3:**
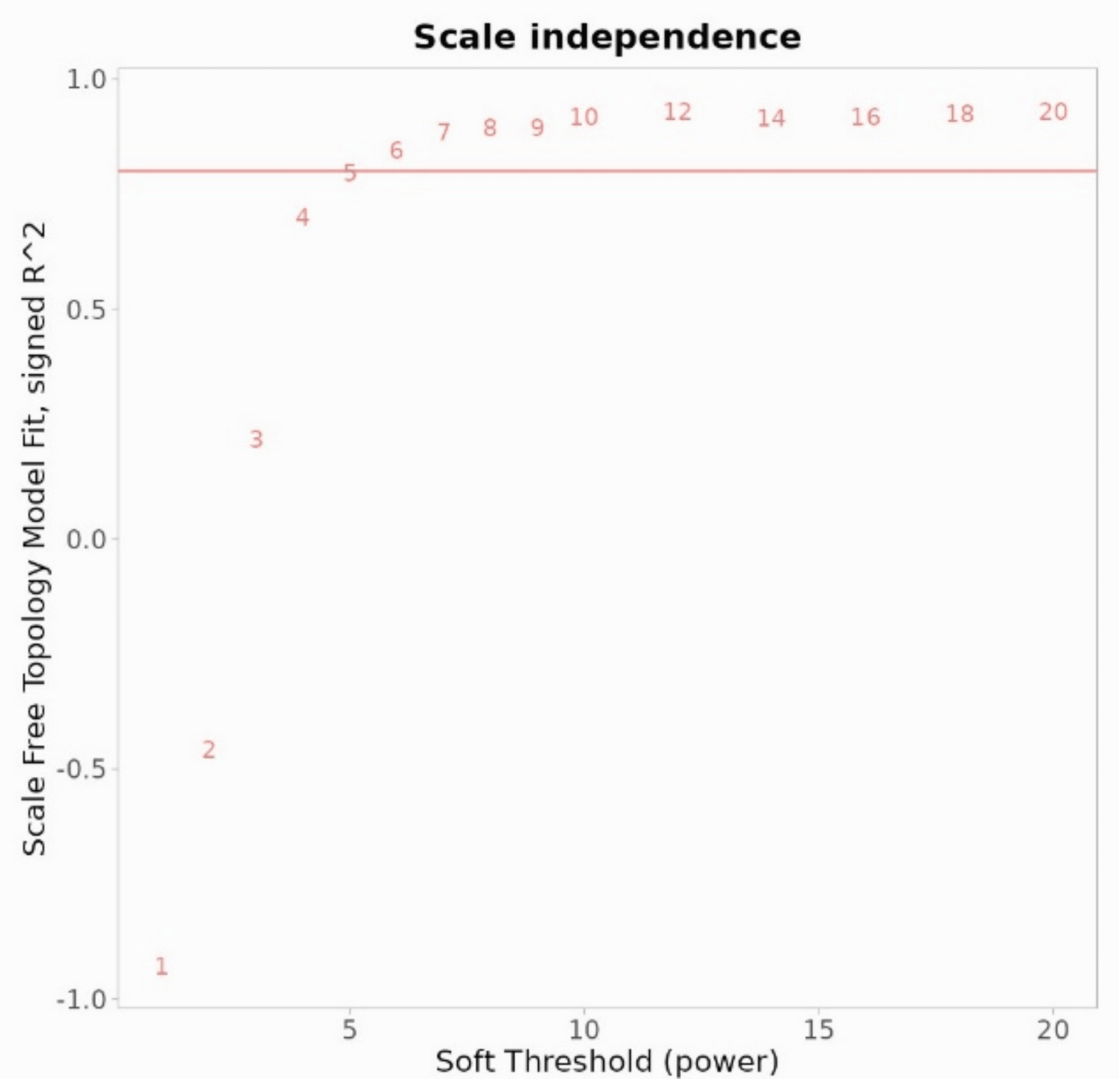
The image shows the soft threshold power of 5 in WGCNA analysis. The soft threshold power of 5 in WGCNA analysis amplifies high correlation values by suppressing weak ones, emphasizing stronger gene interactions. This process mitigates noise impact and enhances the detection of relevant gene modules. The weighted adjacency matrix is then used to construct a topological overlap matrix (TOM), which is then used for clustering algorithms. WGCNA: weighted gene co-expression network analysis

The WGCNA algorithm uses soft thresholding to identify and select relevant gene modules in co-expression networks. A soft threshold power of 5 is used to transform the raw co-expression values into a weighted adjacency matrix, reducing noise and enhancing the transformation of co-expression values as shown in Figure 3.

**Figure 4:**
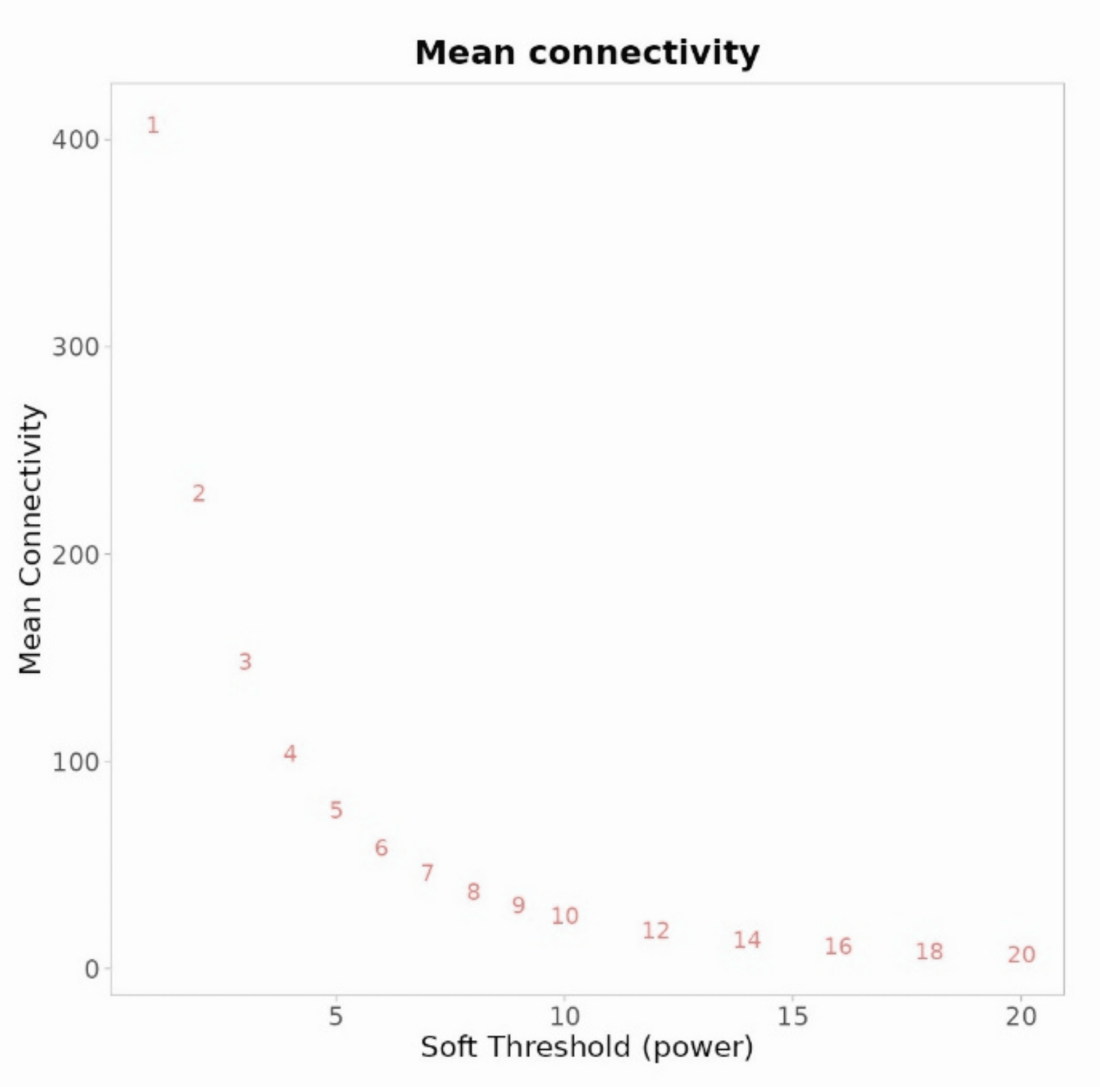
The image shows an analysis of a scale-free topology fitting index R2 and mean connectivity for various soft threshold powers, with an R2 value of 5. The analysis of a scale-free topology involves identifying the optimum soft threshold power for a scale-free network structure, characterized by a few highly connected nodes and many poorly connected nodes. The R2 value measures the degree distribution, while mean connectivity provides insight into the network's overall connectivity and density.

The R2 value and mean connectivity are calculated for each power, with a 5 being a specific threshold. High R2 values indicate a better fit for the desired network structure. This threshold power is used in analysis steps like module detection and functional enrichment to identify relevant gene modules as shown in Figure 4.

**Figure 5:**
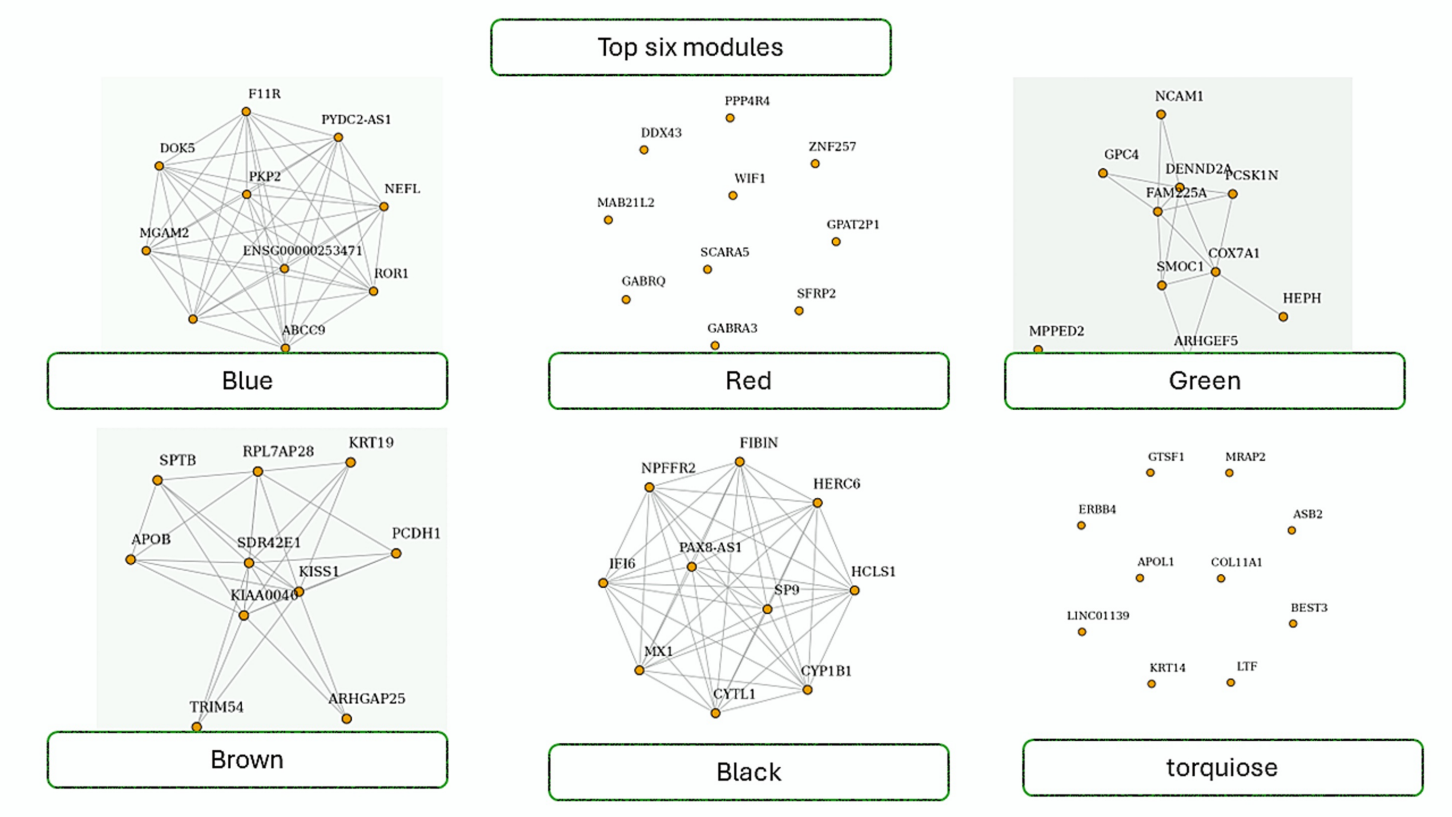
The image shows the top six modules with 10 hub genes from the WGCNA analysis. WGCNA analysis identifies modules as clusters of genes with similar expression patterns, representing biological pathways. Hub genes within modules are crucial for gene network regulation. WGCNA: weighted gene co-expression network analysis

The results of the modules show six network diagrams, each labeled with a different color name (Blue, Red, Green, Brown, Black, Turquoise) and enclosed in a green rectangle. These diagrams represent modules with nodes (circles) and edges (lines connecting the nodes). The nodes are labeled with gene names or identifiers, indicating interactions between elements as shown in Figure 5.

**Figure 6:**
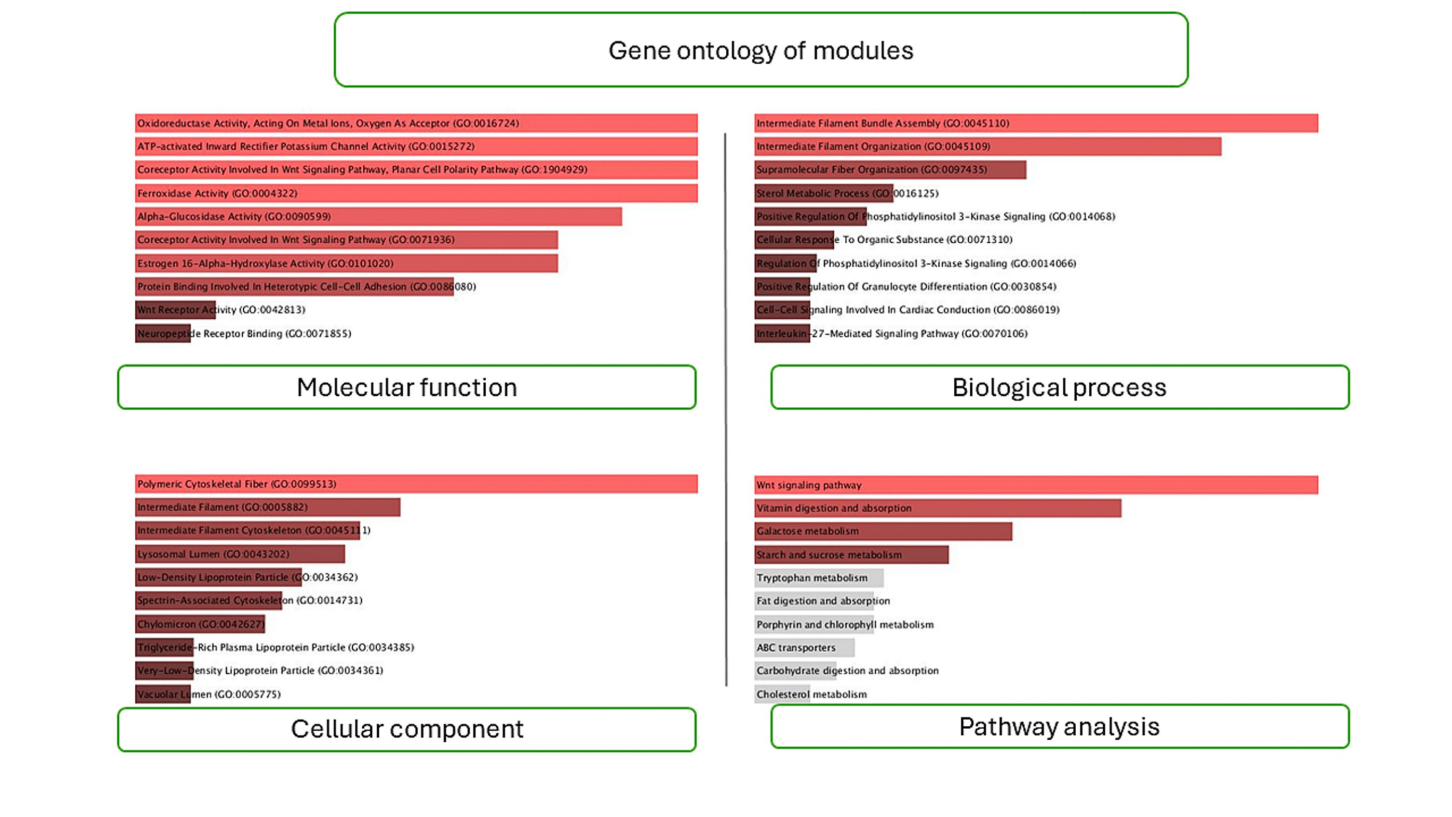
The image shows the gene ontology of all hub genes identified by modules - COL6A1, MMP3, BGN, COL1A2, and FBN2. These genes are considered crucial regulators of periodontal ligament development and remodeling processes, but further research is needed to understand their specific roles and mechanisms. Molecular function focuses on specific tasks performed by gene products, while biological process describes events within a cell or organism. Cellular component identifies the parts of a cell where gene products are active. Gene ontology of modules organizes terms into functional modules based on shared characteristics. Pathway analysis studies the interconnected biochemical reactions and signaling pathways driving cellular processes.

Figure 6 provides a detailed overview of GE modules, which are crucial for understanding gene functions and relationships within an organism. It consists of four categories: molecular function, biological process, cellular component, GE of modules, and pathway analysis.

## Discussion

Wnt paracrine signaling involves secreted glycoproteins interacting with Frizzled receptors to activate multiple pathways, including the well-studied Wnt/β-catenin pathway [17]. In the absence of Wnt signaling, β-catenin is targeted for degradation. When WNT ligands bind to receptors, β-catenin is stabilized, interacts with transcription factors like lymphoid enhancer factor/T-cell factor (LEF/TCF), and activates gene transcription. This process involves various proteins and complexes, ultimately activating target genes [18].

Wnt proteins are traditionally categorized into Wnt1 and Wnt5a classes for their role in Wnt/β-catenin signaling [10,19]. Recent data suggests that specific Wnt-receptor pairings influence signaling outcome rather than Wnt type. Various inhibitors like Dkk, SFRP, and WIF1 regulate the Wnt/β-catenin pathway by interacting with receptors or inhibiting ligand-receptor binding. Intracellular inhibitors like NKD and NLK also modulate pathway activity.

Tooth development involves intricate interactions between dental epithelium and mesenchyme, orchestrated by a signaling network. The process begins with the thickening of oral epithelium, forming a tooth bud that progresses through various stages [4]. Wnt/β-catenin signaling is crucial throughout development, influencing gene expression and tooth formation. Various Wnt genes are expressed at initiation, with distinct roles in epithelial and mesenchymal cells. Wnt signaling, especially the Wnt/β-catenin pathway, is vital for tooth initiation and development, with specific localization in dental epithelium and placodes. Studies have shown that β-catenin, Lef1, and PITX2 play crucial roles in tooth development. PITX2 interacts with Lef1 and ß-catenin, influencing the expression of specific isoforms. In mice, Lef1 gene inactivation halts tooth development at the bud stage, which has implications for epithelial and mesenchymal tissue interactions. LEF1's transient role in epithelium affects gene expression in dental mesenchyme, suggesting a reciprocal communication mechanism. Lef1 deficiency leads to dental epithelial cell loss, while Lef1 overexpression can alter cell fate in developing animals [8,14].

WGCNA is a systems biology method to identify genes with similar expression patterns across different biological conditions or samples. It involves determining the soft threshold power and the connection strength between genes. In this study, the choice of soft threshold power of 5 impacts gene co-expression network sensitivity and specificity, enabling the identification of gene co-expression modules using clustering algorithms like hierarchical or dynamic tree cut, providing insights into biological processes.

WGCNA modules [11,12,20] identified top hub genes COL6A1, MMP3, BGN, and FBN2 (Figures 1-5) in Wnt-based PDL formation. COL6A1, MMP3, BGN, and FBN2 are key genes in the PDL tissue extracellular matrix (ECM). COL6A1 [21] is a type VI collagen component, while MMP3 [22] is involved in matrix degradation in oral diseases. BGN [23] modulates cell-matrix interactions and affects cell behavior in dental mesenchymal stimulation. FBN2 regulates cell signaling and tissue development, possibly modulating Wnt signaling in oral dental papillae. Wnt signaling plays a crucial role in the ECM of the bone marrow (PDL), influencing the synthesis and organization of ECM components such as COL6A1, MMP3, BGN, and FBN2. These interactions ensure the proper formation and maintenance of the ECM, aiding tissue resilience and repair. Wnt signaling can also upregulate MMP3 expression, increasing transcription during tissue remodeling. Biglycan, a proteoglycan that binds to collagen fibrils and other ECM molecules, contributes to the structural integrity of the PDL. Wnt signaling can modulate cellular activities such as proliferation and differentiation, ensuring proper ECM organization. Collagen I, the primary collagen in the PDL, provides structural support and tensile strength. Wnt signaling can enhance the expression of COL1A2, ensuring adequate production of collagen I for forming and maintaining a strong and functional PDL [1,3,24,25].

The genes COL6A1, MMP3, BGN, and FBN2 are crucial for forming and maintaining the ECM of the PDL tissue. COL6A1 encodes the alpha-1 chain of type VI collagen, essential for the PDL's structural integrity and function [26]. MMP-3 is involved in the degradation of ECM components, facilitating the turnover of collagen and other matrix proteins [27]. BGN plays a significant role in collagen fibrillogenesis and the structural integrity of the PDL. FBN2 regulates PDL cell proliferation and differentiation, affecting PDL homeostasis and repair mechanisms. BGN and FBN2 contribute to the PDL's structural and functional integrity, ensuring proper tooth support and periodontal health [28].

Future research should focus on functional validation of hub genes' roles in PDL development, identification of upstream regulators controlling gene expression, and exploring clinical implications for disease management and tissue regeneration in periodontal disease management and tissue regeneration. The study has limitations, including dataset bias, potential gene prioritization, and lack of functional validation. It suggests that additional approaches like GE enrichment and pathway analysis could enhance understanding of hub genes' roles in PDL development.

## Conclusions

This WGCNA study identified six modules and hub genes crucial for PDL formation, including COL6A1, MMP3, BGN, and FBN2. These genes are involved in ECM organization, tissue remodeling, and cell-matrix interactions. Further research could guide therapeutic strategies for periodontal regeneration. The study identified hub genes in the Wnt signaling pathway crucial for PDL formation, offering insights into periodontal development and function. Targeting these genes could enhance tissue regeneration and treatment outcomes, paving the way for further research and therapeutic interventions. The study's bioinformatics approach and lack of functional validation experiments, along with a small sample size, suggest a need for a more comprehensive study.
